# Barriers to exercise and the role of general practitioner: A cross-sectional survey among people with multiple sclerosis

**DOI:** 10.3389/fneur.2022.1016143

**Published:** 2022-11-21

**Authors:** Luca Correale, Luca Martinis, Eleonora Tavazzi, Ludovico Pedullà, Giulia Mallucci, Giampaolo Brichetto, Marco Bove, Michela Ponzio, Paola Borrelli, Maria Cristina Monti, Roberto Bergamaschi, Cristina Montomoli

**Affiliations:** ^1^Department of Public Health, Experimental and Forensic Medicine, University of Pavia, Pavia, Italy; ^2^Multiple Sclerosis Center, IRCCS Mondino Foundation, Pavia, Italy; ^3^Scientific Research Area, Italian Multiple Sclerosis Foundation, Genoa, Italy; ^4^AISM Rehabilitation Center Liguria, Italian Multiple Sclerosis Society, Genoa, Italy; ^5^Section of Human Physiology, Department of Experimental Medicine, University of Genoa, Genoa, Italy; ^6^Laboratory of Biostatistics, Department of Medical, Oral, and Biotechnological Sciences, University G. d'Annunzio Chieti-Pescara, Chieti, Italy

**Keywords:** multiple sclerosis, exercise, physical activity, barriers, general practitioner

## Abstract

**Introduction:**

Regular exercise is strongly recommended for people with MS (pwMS), but recent studies still describe them as sedentary and insufficiently active. The purpose of this study is to identify the major barriers that prevent pwMS from exercising and underline the importance of the general practitioner (GP) in promoting an active lifestyle.

**Materials and methods:**

We performed a multicenter cross-sectional study using a self-administered questionnaire among pwMS. Data about demographics, the disease, current exercise practice, barriers, previous GP's advice to practice, and motivation were collected.

**Results:**

A total of 741 pwMS (age 55.6 ± 12.5 years, 66% females) completed the survey. Most responders (75.3%) did not practice any exercise. Fatigue was the most limiting factor to attending and/or starting an exercise program, followed by travel and/or moving issues, and the lack of time. Only 25.5% of participants received GP's advice to practice exercise, but 48.6% of them attended an exercise program. A greater likelihood of practice was evidenced for people that received the GP's advice than those who had not received it (OR 2.96; *p* < 0.001). Finally, among those who did not practice exercise but received advice from GPs, 69 out of 99 (69.7%) were motivated to start an exercise program.

**Conclusion:**

Fatigue and physical issues are the main barriers to exercise for pwMS, but also other factors not related to the disease seem to be relevant, like travel issues and lack of time. Although few participants received advice to exercise from their general practitioner, his role proved effective in encouraging the practice.

## Introduction

Multiple sclerosis (MS) is a neurological disorder that affects physical and cognitive functions. Typically, the demyelination of the neurons' axons caused by the disease leads to a chronic and increasing level of symptoms and disability (1, 2). To manage the main symptoms of the disease -like fatigue, gait impairment, and depression- researchers recommend engaging in physical activity, i.e., any bodily movement produced by skeletal muscle contraction that results in energy expenditure (3), as a complementary therapy (4, 5). In particular, solid evidence suggests that persons with MS (pwMS) who engage in exercise, defined as a planned activity usually performed repeatedly over time with a specific improvement aim (3), can increase walking autonomy, aerobic capacity, and strength (6–10).

Despite these benefits, recent data showed that most pwMS did not follow the recommendations and persist in sedentary behavior (11, 12). In healthy adults, the reasons for physical inactivity range from personal to environmental factors, but the most common limit is the lack of time due to family and work commitments (13). Conversely, for pwMS, factors preventing exercise are usually related to disease symptoms and disability level (14). However, the disease symptoms and disability greatly vary from person to person, hence the reason for inactivity is frequently a combination of general and disease-related factors, without certainty about which of these has the greatest impact.

Along with rehabilitation, exercise is usually prescribed to pwMS by their specialists. Still, it is often limited in time (commonly, two sessions a week for 6–8 weeks) and circumscribed to a clinical setting. Instead, performing regular activities during leisure-time over an extended period of time in a familiar and stimulating environment could enhance motivation, adherence, and continuity of training. Sports may play a crucial role since they pertain to any form of competitive physical activity aiming at using, maintaining, or improving physical ability and skills while providing enjoyment to participants.

In this context, the role of the general practitioner (GP) could be relevant in order to identify a tailored and effective exercise-promoting strategy. GP is the medical interface that takes care of pwMS after the diagnosis and management at the reference MS Center, when this occurs in age ranges where MS can present, if correctly treated, a slower disability progression (15). Indeed, both pediatric (< 16 years) and late (>40 years) MS onset seem to be associated with a higher risk of unfavorable MS prognosis (16, 17) and are usually directly followed by specialists (most likely, neurologists). The GP can also assist patients in managing comorbidity, which has been associated with delayed diagnosis of MS, progression of disability, lower health-related quality of life, increased MS burden on magnetic resonance imaging, and increased mortality due to MS (18–21).

Concerning physical activity, the GP's support might be crucial to maintain or begin a sports activity in order to enhance spontaneous and continuous exercise training during leisure time. Previous studies found that GP is crucial to stimulate healthy lifestyles in his/her patients, including physical activity, exercise, and sports (22, 23). In the present study, we focused on spontaneous exercise habits (i.e., sports and fitness practice) with the aim to determine the main barriers and the relevance of GP advice in pwMS.

## Methods

### Study design

A multicenter cross-sectional study was conducted using a self-administered questionnaire at the Multiple Sclerosis Center of IRCCS Mondino Foundation in Pavia and at the Italian MS Society (AISM) Rehabilitation Centre Liguria, in Genoa, Italy. The study was voluntarily attended by pwMS ≥18 years old, with a diagnosis of clinically definite MS according to Mc Donald's criteria (24). Exclusion criteria were as follows: severe cognitive impairment or psychiatric disorders impeding or consistently limiting the comprehension of the questionnaire, pregnancy, and disability evaluated *via* the Expanded Disability Status Scale (2) (EDSS) > 9.0.

Data were collected including demographic information, the characteristics of the disease, information about current exercise behavior in terms of sports and fitness practice, previous GP's advice to perform this kind of activities, and motivation to begin a training program. For each person, the aim of the study was explained, and informed consent was signed prior to the involvement in the study. The procedures of the present study were reviewed by the reference Ethical committee for each center (protocol numbers: 20180051956, 12 Jun 2018; 252/2018, 6 May 2019) and performed following the latest revision of the Declaration of Helsinki.

### Characteristics of the disease

To investigate the relationship between the disease and the exercise practice, we collected information about disease phenotype -relapsing-remitting, RR; primary progressive, PP; secondary progressive, SP- and disability level evaluated by a neurologist using the Expanded Disability Status Scale (EDSS) (2).

### Exercise barriers and motivation

The exercise behaviors, barriers, and reasons to abandon previous exercise activities were collected using pointed questions based on the recent literature (13, 14). The questions included the involvement in an exercise program in the last 3 months, the presence/absence of supervision on exercise, the barriers to exercise, and the causes for abandoning previous exercise programs/activities.

To test the hypothesis that GP could be relevant to exercise behavior, we asked them if they received any advice from their GP to begin an exercise program and if they were motivated to start a new exercise program.

### Data analysis

The collected data were analyzed using JASP software (2019, Version 0.11.1). The results are shown as a frequency, mean ± standard deviation, or median (IQR) according to the type of variable. The association between exercise practice or the motivation to start a new exercise program and the GP's advice was analyzed using Chi-square tests. A logistic regression model was used to estimate the odds ratio and the corresponding 95 % Confidence Intervals for practicing exercise considering covariates age, gender, disease duration, GP's advice, level of disability (EDSS as a continuous variable), and disease course. We considered *p* < 0.05 as statistically significant.

## Results

The survey was completed by 741 pwMS (disease duration: 19.7 ± 11.8 years.; mean EDSS 3.6 ± 2.2, median EDSS 3, IQR 2-6), 253 were males and 488 females and the mean age was 55.6 ± 12.5 years (age range: 24–89). The most common disease phenotype was relapsing-remitting (RRMS) (496; 161 males, 335 females; disease duration: 17.1 ± 10.2 years; EDSS 2.6 ± 1.7), followed by secondary progressive (SPMS) (192; 71 males, 121 females; disease duration: 27.8 ± 11.9 years; EDSS 5.9 ± 1.6) and primary progressive (PPMS) (53; 21 males, 32 females; disease duration: 16.3 ± 11.8 years; EDSS 4.8 ± 1.8).

**Figure 1:**
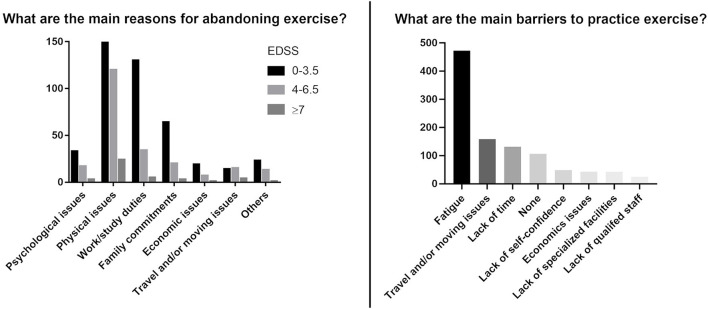
Reasons for abandoning previous exercise activities and barriers to exercise (multiple responses were allowed).

The majority of pwMS (75.3%) did not practice any exercise at the time of the survey, with a higher frequency for SPMS (91.7%) (Table 1). PwMS stated that physical issues related to the disease, and work/study duties, as well as family commitments, were the main reasons to withdraw from previous exercise activities. However, work/study and family commitments were relevant only for lower levels of EDSS (0–3.5), whereas physical issues were the principal reason for abandoning previous activities for higher levels of disability (EDSS ≥ 4; Figure 1).

**Table 1:** Exercise practice and MS course (*n* = 741).

**Do you practice any exercise activities currently?**			
**MS course**	**Yes (%)**	**No (%)**	**Total**
RR	154 (31)	342 (69)	496
SP	16 (8.3)	176 (91.7)	192
PP	13 (24.5)	40 (75.5)	53
Total	183 (24.7)	558 (75.3)	

RR, Relapsing-Remitting; SP, Secondary Progressive; PP, Primary Progressive.

Participants reported fatigue, travel/mobility issues, and lack of time, as the most common barriers to attending and/or starting a new exercise program. Secondary barriers were identified as a lack of self-confidence, economic issues, and a lack of specialized facilities (Figure 1).

Among participants, 189 out of 741 (25.5%) received GP advice to practice exercise and 89 of them (47.1%) actually practiced exercise. Conversely, only 94 out of 552 (17.0%) practiced exercise without having received any advice from the GP (Table 2). Moreover, among those who did not practice exercise but received the advice from GP, 69 out of 99 (69.7%) were motivated to start a new exercise program (Table 2). Interestingly, among pwMS who practiced exercise (183, 24.7%), only 28 (15.3%) were supervised by an exercise specialist.

**Table 2:** General practitioner advice, exercise practice, and motivation to start a new exercise program among those who did not practice exercise.

**GP's advice and exercise practice (*****n*** = **741)**.				
	**Did your general practitioner ever advice you to practice exercise?**			
**Do you practice any exercise activity currently?**	**Yes (%)**	**No (%)**	**Total**	***p***
Yes	89 (48.6)	94 (51.4)	183	< 0.001
No	100 (17.9)	458 (82.1)	558	
Total	189 (25.5)	552 (74.5)	741	
**GP's advice and motivation to start a new exercise program among those who did not practice exercise (n** **=537–21** ***missing*****)**.				
	**Did your general practitioner ever advice you to practice exercise?**			
**Would you be motivated to start a new exercise program?**	**Yes (%)**	**No (%)**	**Total**	***p***
Yes	69 (34.3)	132 (65.7)	201	< 0.001
No	30 (8.9)	306 (91.1)	336	
Total	99 (18.4)	438 (81.6)	537	

Table 3 reports crude and adjusted ORs for the practice of exercise. In the multivariate analysis, people who received GP advice showed about three times higher likelihood of exercise practice (OR 2.96; 95% CI 2–4.4; *p* < 0.001), while increasing disability was associated with about a 20% lower chance of practice (OR 0.78, 95% CI 0.7–0.9; *p* < 0.001), controlling for sex, age, disease duration, and MS course.

**Table 3:** Logistic regression model considering different covariates as predictors of exercise practice.

	**Crude OR**	**95% CI**	***p***	**Adjusted OR**	***p***	**95% CI**
Age (years)	0.96	0.95–0.97	< 0.001	0.98	0.09	0.97–1.00
Disease duration (years)	0.98	0.96–0.99	0.002	1.01	0.26	0.99–1.03
EDSS	0.69	0.63–0.76	< 0.001	0.78	< 0.001	0.69–0.89
Gender (men vs. women)	1.05	0.73–1.49	0.79	0.87	0.50	0.59–1.30
GP's advice (yes vs. no)	4.34	3.02–6.22	< 0.001	2.96	< 0.001	2.00–4.37
MS course (RR vs. SP)	0.20	0.12–0.35	< 0.001	0.60	0.17	0.29–1.24
MS course (RR vs. PP)	0.72	0.38–1.39	0.33	1.64	0.20	0.77–3.49

OR, Odds Ratio; CI, Confidence Intervals.

Dependent variable: Do you attend any physical activity currently? Yes.

## Discussion

Exercise is an important non-pharmacological strategy to improve the quality of life of pwMS, through the increase of physical capacities and autonomy (5, 9, 10). It should be spontaneously and continuously performed by pwMS, according to their possibilities and disability levels, e.g., by practicing tailored sports activities. However, previous studies showed that most pwMS have a sedentary lifestyle and avoid exercise with a negative effect on their health (12, 25, 26). The aims of the present study were to gather information on the current exercise behavior in terms of sports and fitness practice and identify the main barriers to exercise among pwMS. In addition, we investigated if the role of the general practitioner was relevant to motivate pwMS to maintain or begin training programs.

Participants identified disease-related physical issues, work/study duties, and family commitments as the main reasons to have abandoned their previous exercise activities. The “physical issues” were the prevalent reason to abandon exercise for all levels of disability, however, we noticed that for the 0–3.5 EDSS range the work/study duties are also significant. Work/study duties were listed as limiting factors mostly by people with a low level of disability (EDSS < 3.5), and only for a minority of people with high disability levels, that are often prevented from maintaining their professional careers for several issues directly related to MS.

Regarding the reasons for not attending an exercise program at the time of the interview, pwMS declared that the major barrier was fatigue -the most common symptom in MS (27)—followed by travel and/or moving issues, and lack of time. Probably, travel and/or moving issues were not the main cause for abandoning past activities, contrarily to lack of time due to family and work duties, but became more important over time, limiting the practice of new activities.

As we expected most of the sample did not practice any exercise (75.3%), with no GP's advice as a significant predictor. This finding reflects the results of previous studies that showed sedentary behaviors of pwMS, especially in men (25, 28), probably a result of being more physically affected by the disease. Noteworthy, almost all people with SP form (91.7%) stated that they did not attend any physical activity or exercise program. Even though SP is characterized by a progressively increasing disability accrual, studies have shown that exercise is feasible and effective to reduce symptoms and improve fitness and cognition, so it is strongly recommended also in this form (29, 30). Possible reasons for the low rate of pwMS practicing any kind of physical activity are various, applicable to all the disease phenotypes but clearly more pronounced in SP forms, or in pwMS with moderate-to-high disability levels. First, pwMS may have physical issues limiting their ability to drive or use public transportation, rendering them dependent on caregivers to access gyms or structures where physical activities are practiced. Furthermore, these structures might be geographically distant from patients' houses and difficult to reach. In this regard, patients often refer to the onset of symptoms such as spasticity and fatigue associated with the need to maintain a seated position for prolonged periods of time, such as when spending time in a car to reach their destination. This aspect needs to be considered when planning physical exercise, as a gym located at a long distance might represent an issue counteracting the beneficial effect of a physical activity session. Very often fatigue and depression coexist and become more pronounced over time and are related to disability accrual (31), preventing pwMS from performing physical activity on a regular basis. A higher level of disability further limits the possibility to exercise regularly as requires a professional intervention from physiotherapists, in the context of neuromotor rehabilitation.

According to our findings, pwMS receiving GP advice to practice exercise are more likely to attend an exercise program. Similarly, pwMS not practicing exercise but receiving advice were more motivated to start an exercise program. These results confirm the positive role of the GP in promoting exercise in pwMS, as previously demonstrated in older adults (23). Moreover, as stated in a study by Canning et al., people that were directly referred to an exercise program by the GP were more likely to adhere to the Canadian Physical Activity Guidelines for Adults with Multiple Sclerosis (PAGs) (22).

Considering these results, the GPs could have a fundamental role to reduce the physical inactivity of their patients. Fatigue is known to be the most limiting factor to practicing exercise but was also shown that regular exercise is effective to reduce fatigue and other related physical symptoms. Our results underline the key role of GP in promoting a positive lifestyle in their patients, including recommendations on physical activity, exercise, and sports. Finally, only 28 pwMS practicing exercises (15.3%) were supervised by a specialist. The lack of supervision by an expert trainer could be dangerous for pwMS due to incorrect management of exercise intensity. Similarly, in this case, general practitioners can recommend pwMS not to self-administer exercise, in order to reduce injury risks and maximize exercise benefits.

Although the findings of this study are consistent with the literature, some limitations are noteworthy. The cross-sectional design does not allow comparing exercise barriers before and after the onset of the disease. The answers were self-reported, and we did not record the amount of physical activity or exercise. Similarly, we have no information about the advice of the general practitioner, such as the number and timing of the advice, the exact words, and/or messages delivered to patients.

For these reasons, more studies are needed to understand the underlying mechanisms limiting pwMS to spontaneously practice regular exercise. The role of sports should also be further investigated, focusing on the type and modality of the most commonly practiced and ceased activities in order to help clinicians, e.g., general practitioners, to suggest tailored and effective exercise-promoting strategies. Our findings confirm that fatigue and lack of time due to work/family commitments are the main barriers and new exercise strategies -for example, tele-exercise- need to be considered. Finally, we observed that the role of GP is crucial to persuade pwMS to practice exercise and sports. Therefore, the general practitioners are encouraged to suggest an exercise program for pwMS who are not currently exercising.

## Data availability statement

The raw data supporting the conclusions of this article will be made available by the authors, without undue reservation.

## Ethics statement

The present study was reviewed and approved by IRB Fondazione IRCSS San Matteo. The patients/participants provided their written informed consent to participate in this study.

## Author contributions

LC, LM, GB, MB, MP, and RB: conceived and designed the study. LM, LP, GM, GB, MB, and MP: performed the study. CM, MM, PB, and LC: analyzed the data. LC, LM, ET, LP, and GM: wrote the manuscript. RB, CM, GB, and MB: reviewed the manuscript. All authors contributed to the article and approved the submitted version.

## Funding

This study was supported by FISM Grant 2017/R/4 for the Reasearch Project Costs of comorbidity in multiple sclerosis people.

## Conflict of interest

The authors declare that the research was conducted in the absence of any commercial or financial relationships that could be construed as a potential conflict of interest.

## Publisher's note

All claims expressed in this article are solely those of the authors and do not necessarily represent those of their affiliated organizations, or those of the publisher, the editors and the reviewers. Any product that may be evaluated in this article, or claim that may be made by its manufacturer, is not guaranteed or endorsed by the publisher.
